# Perceptions and drivers of healthcare provider and drug dispenser practices for the treatment of malaria in pregnancy in the context of multiple first-line therapies in western Kenya: a qualitative study

**DOI:** 10.1186/s12936-023-04698-w

**Published:** 2023-09-14

**Authors:** Caroline B. Osoro, Stephanie Dellicour, Eleanor Ochodo, Taryn Young, Feiko O. ter Kuile, Julie R. Gutman, Jenny Hill

**Affiliations:** 1 Centre for Global Health Research, Kenya Medical Research Institute, P.O. Box 1578, Kisumu, 40100 Kenya; 2 Division of Epidemiology and Biostatistics, Department of Global Health, Faculty of Medicine and Health Sciences, Stellenbosch University, P.O. Box 241, Cape Town, 8000 South Africa; 3 Department of Clinical Sciences, Liverpool School of Tropical Medicine, Pembroke Place, Liverpool, Liverpool, L3 5QA UK; 4 Malaria Branch, Division of Parasitic Diseases and Malaria, U.S. Centers for Disease Control and Prevention, 1600 Clifton Road, Atlanta, GA 30333 USA

**Keywords:** Anti-malarials, Artemisinin-based combination therapies, Malaria, Pregnancy, Healthcare providers, Knowledge, Behaviour, Health practices, Kenya

## Abstract

**Background:**

Emergence of *Plasmodium falciparum* resistance to artemether-lumefantrine in Africa prompted the pilot introduction of multiple first-line therapies (MFT) against malaria in Kenya, potentially exposing women-of-childbearing-age (WOCBAs) to anti-malarials with unknown safety profiles in the first trimester. This qualitative study explored knowledge and perceptions among healthcare providers providing malaria treatment to WOCBAs and pregnant women.

**Methods:**

In-depth interviews were conducted with purposively selected public and private health facility (HF) and drug outlet (DO) providers within and outside the pilot-MFT area. County health managers were interviewed about their knowledge of the national treatment guidelines. Transcripts were coded by content analysis using the World Health Organization health system building blocks (leadership/governance, financing, health workforce, health information systems, access to medicines, and service delivery).

**Results:**

Thirty providers (HF:21, DO:9) and three health managers were interviewed. Eighteen providers were from HFs in the pilot-MFT area; the remaining three and all nine DOs were outside the pilot-MFT area. The analysis revealed that providers had not been trained in malaria case management in the previous twelve months. DO providers were unfamiliar with national treatment guidelines in pregnancy and reported having no pregnancy tests. Health managers were unable to supervise DOs due to resource limitations. Providers from HFs and DOs noted poor sensitivity of malaria rapid diagnostic tests (RDTs) and hesitancy among patients who associated malaria-RDTs with HIV testing. Almost all providers reported anti-malarial stock-outs, with quinine most affected. Patient preference was a major factor in prescribing anti-malarials. Providers in HFs and DOs reported preferentially using artemether-lumefantrine in the first trimester due to the side effects and unavailability of quinine.

**Conclusion:**

Knowledge of malaria case management in drug outlets and health facilities remains poor. Improved regulation of DO providers is warranted. Optimizing treatment of malaria in pregnancy requires training, availability of malaria commodities, and pregnancy tests.

## Background

About 89% of all pregnancies in the World Health Organization (WHO) Africa region (AFRO) were at risk of malaria infection, and 32% had an infection during pregnancy in 2021 [1, 2]. Malaria in pregnancy is associated with severe maternal anaemia, maternal mortality, pregnancy loss, preterm delivery, and low birth weight [3–5]. In malaria-endemic areas, the WHO recommends a three-pronged approach, including case management, prevention using long-lasting insecticide nets, and, for HIV-negative women, intermittent preventive treatment of malaria in pregnancy (IPTp) using sulfadoxine-pyrimethamine in the second and third trimesters [6]. Not all anti-malarials are safe for pregnancy, especially in the first trimester. It is essential that healthcare providers know and adhere to malaria case management guidelines for women-of-childbearing-age (WOCBA) and pregnant women to ensure effective and safe treatment [7].

Malaria in Kenya accounts for an estimated 13–15% of outpatient consultations [8]. Most cases are reported in the Lake endemic zone, where in 2020, 29% of children under 5 years were treated for fever compared to 10% in the low-risk zone [8]. The 2019–2023 National Malaria Control Programme’s Strategic Framework in Kenya seeks to treat all suspected malaria cases according to the national malaria treatment guidelines [9].

Parasitological diagnosis is recommended in all pregnant women with a fever. However, presumptive symptomatic treatment can also be offered if malaria rapid diagnostic tests (RDTs) or microscopy are unavailable [10]. At the time of this study, the recommended treatments for uncomplicated malaria in pregnancy were oral quinine (first trimester) and artemether-lumefantrine (AL, second and third trimesters), and parenteral artesunate for severe malaria (all trimesters) [10]. In 2013, a study in Kenya’s Lake endemic zone reported correct malaria case management practice in pregnancy in only 45% of health facilities and 31% of drug outlets [11]. In the same study, 93% of healthcare providers tested for malaria or accurately described signs and symptoms consistent with clinical malaria in the general population, with 77% providing microscopy at health facilities compared to 5% at drug outlets [12]. While several studies have shown improvements in case management practices for WOCBA and pregnant women at health facilities with access to job aids and guidelines, healthcare providers in drug outlets still lag behind [13–16].

The emergence of *Plasmodium falciparum* resistance to artemisinin-based combination therapy (ACT) in Southeast Asia, Rwanda, and Uganda is a concern for East Africa [17–22]. Since 2006, AL has been made widely available and is free of charge in government-run health facilities in Kenya [23–26]. The WHO recommends diversifying artemisinin-based combinations as a strategy to combat the development of anti-malarial drug resistance [27]. In June 2020, the Kenyan Ministry of Health embarked on a pilot study on the feasibility of rotational multiple first-line therapies (MFT) for malaria in Homa Bay county (Pers. commun., Kokwaro, 2023). The introduction of MFT is likely to increase the exposure of women in early pregnancy to the newer generation of artemisinin-based combinations not yet recommended for use in the first trimester because WOCBA are not routinely screened for pregnancy and do not know or do not report they are pregnant, and are therefore treated with the same drugs used in non-pregnant adults. In addition, in 2021, a prospective, observational study (MiMBa pregnancy registry) was launched in Homa Bay to generate more evidence on the safety of the other artemisinin-based combinations used in the MFT pilot, including pyronaridine-artesunate, dihydroartemisinin-piperaquine, amodiaquine-artesunate (Clinicaltrials.gov registration NCT04825782).

The study sought to explore knowledge and perceptions among healthcare providers providing malaria treatment to WOCBA and pregnant women to determine awareness of malaria diagnosis and treatment regimens in different trimesters in the context of the Kenyan Ministry of Health MFT pilot in Western Kenya.

## Methods

### Study sites

The study was conducted from March to April 2022 in Homa Bay County in Western Kenya, located along the shores of Lake Victoria. Malaria transmission is perennial and holo-endemic, with peaks following the two rainy seasons, March through May and October through December. In collaboration with private partners, the government piloted a rotational MFT project in all government and not-for-profit health facilities in the County between June 2020 and September 2022. Between June 2020 and March 2021, AL was used as first-line treatment for uncomplicated malaria in adults (excluding pregnant women) and children over 24 months in all sites. Between April and November 2021, AL was replaced by dihydroartemisinin-piperaquine as the first-line treatment on the mainland and by pyronaridine-artesunate on Mfangano and Rusinga Islands. Amodiaquine-artesunate replaced AL as first-line treatment on the mainland between July and September 2022.

After the pilot period, AL was the first-line treatment for uncomplicated malaria. The recommendation was to continue treating pregnant women according to the national treatment guidelines (AL in the second and third trimesters, in the first trimester, 7 days of oral quinine or AL if quinine is unavailable). The government conducted training of heads of public and faith-based health facilities in the study area. The trainees were expected to train other healthcare providers in their facilities and community health volunteers through a series of sensitization meetings. The MiMBa study operated in 41 public and private health facilities in the MFT pilot area, provided training to healthcare providers in antenatal, delivery and postnatal clinics, and study staff performed early pregnancy ultrasounds to study participants attending antenatal care (ANC) clinics. No training on case management was provided as part of this observational study.

### Participants

In-depth interviews were conducted with healthcare providers and drug dispensers purposively selected from dispensaries, health centres, hospitals, and drug outlets, comprising government, private, and faith-based facilities. The study aimed to sample 30 healthcare providers and 3 health managers to achieve anticipated data saturation. Participants were selected from 14 health facilities and eight drug outlets in Homa Bay County, including Mfangano Island and Rusinga Island, randomly selected from a list compiled by community health volunteers who were asked where anti-malarial drugs were sold. A drug outlet is authorized to perform simple diagnostics such as pregnancy tests and rapid malaria diagnosis in addition to selling drugs. In each drug outlet, the person available on the day of the study team visit was interviewed. In health facilities, between one to four healthcare providers responsible for seeing adult outpatients or working at the pharmacy were interviewed. Interviews were conducted every day of the week, and participants were reimbursed for their time.

### Data collection

Two trained and experienced social scientists (IA-female and BO-male) conducted face-to-face interviews in English, each lasting approximately 60 min. Both interviewers had been trained in interview techniques, IA had no prior relationship with the participants, while BO was a field officer for the MiMBa study in 11 facilities where 18 of the healthcare providers worked. Interviews were conducted privately near the participant’s place of work, and field notes were not used. Interviewers used a pilot-tested topic guide encompassing: (1) anti-malarial efficacy; (2) safety; (3) tolerability; (4) regimen (duration) and packaging; (5) patient preferences and perceived adherence; (6) personal preferences; (7) availability; (8) cost considerations; (9) access to training or information; (10) pregnancy testing in women of childbearing age; (11) any other clinical considerations when prescribing.

In addition, study team members interviewed three health managers involved in malaria control, one from each sub-county in Homa Bay (Suba North sub-county and Homa Bay Town sub-county) and one from the Homa Bay county health management team. Health managers were interviewed regarding (1) training on treatment guidelines; (2) familiarity with guidelines for pregnancy testing and malaria treatment in pregnancy; (3) anti-malarial availability and procurement; (4) regulation of drug outlets; (5) monitoring healthcare provider adherence to guidelines. No repeat interviews were done.

### Data management and analysis

Interviews were audio-recorded and transcribed verbatim by experienced study team members. Transcripts were not returned to participants for corrections or clarification. Healthcare providers were assigned a unique identification number in transcripts to protect their identity. Transcripts were transferred to NVivo-12 for coding and analysis by CBO. Themes were coded by content analysis using the WHO health system building blocks (finance, leadership/governance, health information, health workforce, products and technologies/access to essential medicines, and service delivery) [28]. Inductive coding allowed emergent themes and sub-themes to be added to each building block in the coding framework. Four research team members discussed themes (CBO, JH, SD and JG), and consensus on any differences in thematic coding was reached through discussion. The analysis explored differences in knowledge and perceptions between healthcare providers in health facilities and drug outlets and within and outside the MFT pilot and MiMBa project.

### Ethical considerations

This study was approved by the Kenya Medical Research Institute’s Scientific and Ethics Review Unit (4277), the Stellenbosch University Health Research Ethics Committee (S21/03/056), the Liverpool School of Tropical Medicine (LSTM) Research Ethics Committee (21–049). CDC Human Research Protections Office reviewed and determined that CDC participation did not meet the definition of engagement in this study. Participants provided written informed consent. The study has been reported following the Consolidated criteria for reporting qualitative research (COREQ) guidelines [29].

### Patient and public involvement

Community health volunteers (CHVs) in the study area were questioned to determine the health facilities and drug outlets that sold anti-malarials. CHVs are lay members of the community who work either for pay or as volunteers in association with the local healthcare system in urban and rural environments. CHVs share ethnicity, language, socioeconomic status, and life experiences with the community members they serve and typically serve where they live [30].

## Results

### Participant characteristics

Thirty healthcare providers and three health managers were interviewed; 18 (55%) were women. There were no refusals. Of 21 healthcare providers from health facilities, 13 were from government-owned facilities, 4 were from private facilities, and 4 were from faith-based facilities. Nine providers were from drug outlets. Of 21 health facility respondents, 16 were from facilities involved in both the MFT pilot and MiMBa project; two were involved in MiMBa, one was involved in the MFT pilot, and two were involved in neither. None of the drug outlet providers were involved in either project. All providers held two-year diploma certificates except for three degree holders (one nurse, one clinical officer, and one health manager), one with a high school diploma, and one with a one-year nursing certificate. One provider had a diploma in education. Respondents had varying lengths of service at their facilities ranging from a few weeks to ten years (Table 1).

**Table 1:** Characteristics of health facilities and drug outlet respondents

Facility type	Facility	Number of respondents	Gender	Cadre	Qualification	Time of service in the facility	MiMBa Pregnancy registry study site	Multiple first line antimalarial project site	Both MiMBa registry and multiple first-line project
*Health facilities*									
Private-run	Facility 1	1	Male	Pharmacist	Diploma	Less than a month	**×**	**×**	**×**
	Facility 2	1	Female	Clinical officer	Diploma	1.5 years	**√**	**×**	**×**
	Facility 3	1	Female	Nurse-in charge	Degree	1 year	**×**	**×**	**×**
	Facility 4	1	Male	Nurse	Diploma	3 months	**√**	**×**	**×**
Faith-based	Facility 5	1	Male	Clinical officer	Diploma	6 years	**×**	**√**	**×**
	Facility 6	3	Female	Clinical officer	Diploma	2 months	**√**	**√**	**√**
			Female	Clinical officer	Diploma	7 months	**√**	**√**	**√**
			Female	Nurse	Diploma	9 months	**√**	**√**	**√**
Government-run	Facility 7	2	Female	Clinical officer	Diploma	7 years	**√**	**√**	**√**
			Male	Clinical officer	Diploma	6 years	**√**	**√**	**√**
	Facility 8	1	Female	Nurse	Diploma	4 years	**√**	**√**	**√**
	Facility 9	1	Female	Clinical officer	Diploma	1.5 years	**√**	**√**	**√**
	Facility 10	1	Male	Clinical officer	Degree	–	**√**	**√**	**√**
	Facility 11	4	Female	Clinical officer	Diploma	2 years	**√**	**√**	**√**
			Male	Clinical officer-in charge	Diploma	3.5 years	**√**	**√**	**√**
			Male	Pharmacist	Diploma	2 years	**√**	**√**	**√**
			Female	Clinical officer	Diploma	2 years	**√**	**√**	**√**
	Facility 12	1	Female	Clinical officer-in charge	Diploma	4 years	**√**	**√**	**√**
	Facility 13	1	Male	Nurse-in charge	Diploma	4 years	**√**	**√**	**√**
	Facility 14	2	Male	Pharmacist	Diploma	3 years	**√**	**√**	**√**
			Male	Clinical officer-in charge	Diploma	5 years	**√**	**√**	**√**
Subtotal		21							
Drug outlets	Drug outlet 1	1	Male	Nurse aid	Certificate	10 years	**×**	**×**	**×**
	Drug outlet 2	1	Female	Pharmacist	Diploma	2 months	**×**	**×**	**×**
	Drug outlet 3	1	Female	Nurse	Diploma	6 months	**×**	**×**	**×**
	Drug outlet 4	2	Female	Pharmacist	Diploma	1 year	**×**	**×**	**×**
			Female	Pharmacy assistant	High school leaver	10 months	**×**	**×**	**×**
	Drug outlet 5	1	Female	Salesperson	Diploma in education	1 month	**×**	**×**	**×**
	Drug outlet 6	1	Male	Clinical officer	Diploma	2 years	**×**	**×**	**×**
	Drug outlet 7	1	Male	Nurse	Diploma	2 years	**×**	**×**	**×**
	Drug outlet 8	1	Male	Pharmacist	Diploma	10 years	**×**	**×**	**×**
Subtotal		9							
*Health managers*									
	County health management team	1			Degree	2 years			
	Sub-county health management team	2			Diploma	5 years			
					Higher diploma	8 years			
Subtotal		3							
Total		33							

### Themes

The key emergent themes on the management of malaria in pregnancy are presented here by each WHO building block. They are summarized in Table 2, together with the implications of each theme for the national programme.

**Table 2:** Implications of findings for malaria control programmes

Health system building block	In-depth interview theme	Implications for programmes
Health workforce	Perceptions on the treatment of malaria in pregnancy	Positive provider perception is favourable for the programme
	Knowledge of malaria’s effects on pregnancy	Training needed to increase knowledge
	Training in malaria in pregnancy	Training is required for both health facility and drug outlet providers
Leadership/Governance		
	Knowledge of national malaria management guidelines	Dissemination is required, especially for healthcare providers in drug outlets and private health facilities not involved in the multiple first-line treatment project
	Monitoring health worker compliance with malaria management guidelines	Priority to be given to monitoring of drug outlet providers
Service delivery		
	Diagnosis of malaria	Healthcare providers explain to patients how rapid diagnostic tests work and emphasize the difference with an HIV test kit, a broader social and behavioural change strategy to increase test acceptance
		Provide adequate rapid diagnostic test kits and gloves to health facilities
		Educate on infection prevention measures targeting needlestick injuries
		Training providers to emphasize parasitological diagnosis of malaria and sensitivity of diagnostic test kits
	Assessing for pregnancy	Regular supervision of drug outlets to ensure the availability of pregnancy test kits
	Drug stock-outs	Ensure regular supply of anti-malarials by timely procurement and redistribution to health facilities as needed
Products and technologies or access to essential medicines		
	Antimalarial drug characteristics	The change in WHO recommendation from quinine to artemether-lumefantrine for the treatment of first-trimester uncomplicated malaria means that the preference for artemether-lumefantrine over quinine is no longer contraindicated
	Factors considered when prescribing anti-malarials	Drug cost and patient preference as factors in prescription imply that some prescribing practices may contravene guidelines. Regular supervision to ensure recommended anti-malarial drug availability in health facilities and drug outlets
	Challenges of having different drug recommendations	Copies of national guidelines and job aids should be available at health facilities and drug outlets. Regular training of health care providers on current malaria guidelines should be done

### Health workforce

#### Perceptions on malaria treatment in pregnancy

Healthcare providers in health facilities and drug outlets generally had no reservations about giving anti-malarials to pregnant women. Factors considered in prescribing anti-malarials were trimester, the severity of illness and regimen, i.e., mode of administration, number of tablets, and number of days. The national guidelines recommending different drugs for treatment in the first vs. other trimesters were considered impractical by some providers in health facilities and drug outlets. Providers in both health facilities and drug outlets reported using artemether-lumefantrine instead of quinine in the first trimester. This issue was also identified by a health manager who felt that having quinine as a first-line anti-malarial in the first trimester was ineffective as it was usually out-of-stock.

*“The guideline says the first line of management is quinine to pregnant mothers. But you would hardly find quinine in the facilities. So the guideline can say it’s quinine, but we have other drugs that we can give. So it is a challenge. Following the guideline but on the ground, things are different.* " *(R7, Health management team)*.

#### Knowledge of malaria in pregnancy

All but one respondent, a drug outlet provider, could name at least one complication of malaria in pregnancy. Complications mentioned were maternal anaemia and death, preterm labour, decreased appetite, fever, fatigue and infant outcomes including miscarriages, stillbirths, intrauterine growth retardation, preterm and low birth weight babies, and malaria in newborns.

#### Training in the last 12 months

Only a few healthcare providers reported having received malaria in pregnancy training in the twelve months preceding the interview. Except for facilities involved in MFT or MiMBa, none of the private health facilities or drug outlet providers had received training.

### Leadership/governance

#### Knowledge of national malaria treatment guidelines

Unlike drug outlets, most health facility providers demonstrated knowledge of the national malaria management guidelines. They also had greater awareness that trimester should be considered when prescribing anti-malarials to pregnant women. None of the respondents from the private health facilities that were not involved in either MFT or MiMBa demonstrated any knowledge of the national guidelines.

#### Monitoring health worker compliance with malaria management guidelines

Health managers reported continuous supervision of health facilities but that this was dependent on the availability of funds. One manager claimed to conduct spot checks at drug outlets, while another explained that monitoring drug outlet practices was only a recent development. The three managers reported challenges with monitoring drug outlets for compliance, citing a lack of funds and the non-receptivity of health providers.

*“We assess this through supervision, through on-job training, through mentorship that you conduct like every month, at times quarterly depending on the availability of the funds”. (R7, Health management team)*

*“So, the question they ask, “Why should you come and do a DQA on me or giving me support supervision; yet you don’t supply me with anything?“ (R21, Health management team)*.

### Service delivery

#### Diagnosis of malaria

Knowledge of the symptoms of malaria was generally good among providers in health facilities and drug outlets, and both reported using rapid diagnostic tests or microscopy to make a diagnosis. It emerged, however, that some providers in both groups offered presumptive treatment based on clinical symptoms. Reasons for this included a patient’s inability to pay for laboratory testing, refusal to be tested, or a negative test result in a symptomatic patient. A few providers reported cases of needle stick injuries while drawing blood. Another challenge to testing was a lack of gloves.

Some providers had encountered clients who refused a rapid diagnostic test for malaria as they assumed it was a test for HIV or a sexually transmitted infection, one indicating that clients preferred microscopy.

*“Because the rapid test kits came sometimes back so they realise that when they see this, someone can think now we are doing a test for HIV. So, majorly the fear of the unknown like now this is HIV I am going to be tested. Someone can say no”. (R19, health facility).*

Poor sensitivity of rapid diagnostic tests was widely reported as a disadvantage compared to microscopy. Some providers attributed this to the tests being unable to detect malaria species, and others due to them being unable to show parasite density and, therefore, the severity of malaria.

*“it doesn’t indicate the severity of malaria. It only says it is positive, it is negative. But for the microscopy, it will show the number of strains that are there. How many pluses. Now it will help you differentiate the severity of the malaria.“ (R23, health facility)*.

Availability of malaria rapid diagnostic tests was a challenge across all types of facilities, with one provider reporting encountering an expired rapid test kit.

#### Assessing for pregnancy

Healthcare providers generally assessed for pregnancy using the last menstrual period date and pregnancy detection tests. Lack of pregnancy tests in drug outlets meant some drug dispensers had to refer clients elsewhere.

#### Anti-malarial of choice for treatment in pregnancy

When asked for their drug of choice for malaria in pregnancy, regardless of trimester, most providers named intravenous artesunate due to its ease of administration, quick action and short regimen.

*“would prefer these mothers being given injectables. That is the Artesunate. It is easy to administer and the duration is also not taking a number of days. You know artesunate is given intravenously so I think it also works faster” (R10, health facility)*.

Some providers preferred artemether-lumefantrine due to its availability and safety in pregnancy, while a few preferred the newer artemisinin-based combinations, dihydroartemisinin-piperaquine and pyronaridine-artesunate, due to the regimen of once-daily dosing for three days.

#### Drug stock-outs

Healthcare providers from drug outlets and health facilities mentioned sending clients to buy drugs elsewhere due to stock-outs. To counter this, drugs were redistributed across facilities, or facility improvement funds were used to procure anti-malarials before government sent stock. A common concern amongst providers was the stock-out of quinine. Some providers from drug outlets stated that intravenous and oral quinine were slow-moving commodities and did not see a need to stock them.

*“We have not been getting prescriptions for it. That is why the latest stock we had, after selling them, we said no. Because of the low demand. Because when you bring it there, some got expired.“ (R30, drug outlet)*.

### Products and technologies or access to essential medicines

#### Anti-malarial drug characteristics

Most providers described patients complaining that quinine had a bitter taste and caused ringing of the ears (tinnitus). A few also noted that quinine was associated with premature contractions, and they prescribed salbutamol concurrently to counter this.

“*Quinine is good when you are monitoring the patient in the hospital because quinine, they cause contractions in women. And when we are monitoring here, we combine quinine with salbutamol to avoid those contractions.“ (R13, health facility).*

A few providers explained that AL had a sour taste and smell and caused nausea and vomiting, while some said injectable artesunate had no side effects. On the packaging, providers preferred ACT blister packs over quinine packaging. They felt that dispensing quinine into sachets and envelopes by hand was unhygienic and could lead to losing pills.

“*You see like for the quinine that we have been using, they are packaged in a cloth. So, you keep on, you pick one, one. Either while dispensing, even when the client is taking the ones, the one, one, some can fall down.“ (R02, health facility).*

It emerged that blister packs were easier to dispense and increased adherence as they were well-labelled.

“*AL. It is nice. The way it is four-four and even they write day one, day two…very nice, the blister pack. So it guides you.“ (R11, health facility).*

Providers suggested the oral quinine regimen of three times daily for seven days was too long; this, in addition to side effects, contributed to poor adherence. The counting of drops during the administration of intravenous quinine was considered tedious by one provider. A few providers found injectable artesunate’s three doses in twenty-four hours more favourable. While some providers noted that AL had a significant pill burden, with four tablets taken twice daily for 3 days, most preferred the AL regimen over oral quinine because of the shorter number of treatment days.

#### Factors considered when prescribing anti-malarials

Drug safety in pregnancy was an important consideration in the prescription of anti-malarials, according to most providers in both health facilities and drug outlets.

“*One of the things you need to know about the drug is how safe is that drug to this pregnant mother”* (*R10, health facility*).

Most providers cited drug cost as a consideration when prescribing anti-malarials. A unique and worrying perspective from a drug outlet provider was that he would sell half the required dose if the client could afford that.

“*Some may not be able to buy the whole dose of the antimalarial. So what I do, I tell her to take at least a half of the dose, that is one and a half days. Then after that she comes back and adds another.“ (R33, drug outlet).*

It emerged that patient preference was a consideration in anti-malarial prescription in health facilities and drug outlets. Providers explained that patients preferred injectables (artesunate injection) over oral drugs and AL over quinine. A unique perspective from a health facility provider was that some women, to avoid taking quinine, would opt to wait until it was safe to take AL in the second trimester.

“*Some of them nowadays know, they’ll say, “Ah, I am waiting for that period when you’ll tell me that it’s okay for me to take AL.“ Or they will buy out over the counter.“ (R25, health facility)*.

A few health facility providers did not think that patient preference was a factor when prescribing anti-malarials.

*“It’s me to convince the patient on the choice of drug to take.“ (R16, health facility).*

*“I will not be swayed by the client preference. I’ll give the correct thing according to the guideline.“ (R19, health facility)*.

It emerged that patient adherence was another factor considered in anti-malarial prescribing. To increase adherence, one provider reported giving the first dose of AL as directly observed therapy (DOT) and the remaining doses to be taken at home.

Other factors considered were drug availability and expiration dates, drug allergies, patient weight and age, and comorbidities like hypertension, HIV, and diabetes.

#### Challenges of different drug recommendations in different populations

Some providers noted that, despite the government MFT pilot, there were fewer drugs for use in pregnancy.

***“****I feel that the drugs for those who are not pregnant are more than for the pregnant women. So, the pregnant women feel left behind. So, they should bring more drugs for the pregnant women; so, that they also feel better.****”****(R5, drug outlet)*.

It emerged that there was confusion about which drug was to be used in which population.

*“Most people are confused on what should be given to certain age groups, the pregnant women. So, the information was not a bit clear.“ (R04, health facility)*.

One provider mentioned a lack of job aids as a challenge in correctly prescribing the MFT drugs.

*“The only challenge comes in when the drugs are new in the environment. New in the environment means when the clinicians are not conversant with the new molecules, you have to make sure that they know that this drug exists and you need to have job aids apart from sensitisation. They need to have their job aids besides them. Because basically there’s this phobia of this is a new drug I’ve not prescribed. You have not known much about it. What will happen because it’s my first time prescribing it. The more you practice, the more you get acquainted with the drug, the more you get eager to know what happens.” (R20, health facility).*

## Discussion

Healthcare providers in Western Kenya lack basic knowledge of national malaria treatment guidelines and training in malaria case management, including for WOCBA and pregnant women. This was particularly evident in private sector facilities and drug outlets. In addition, poor regulation and monitoring of case management practices, widespread drug stock-outs, and poor availability of pregnancy detection kits were noted. Healthcare providers generally preferred artemether-lumefantrine (AL) and injectable artesunate over quinine when prescribing anti-malarials in the first trimester due to quinine’s side effects, long regimen and packaging, patient preference and adherence.

Despite the problem of training and monitoring private health facilities and drug outlets being identified a decade ago, there continues to be a lack of resources dedicated to their regulation [31]. Significant knowledge gaps were noted among providers in drug outlets. In addition, some drug outlet providers did not have medical training, raising concerns about the need for supervision or oversight. This was reported by the health managers, who explained that drug outlet training and monitoring was a recent activity that was not well funded and poorly received. Drug outlet providers did not understand why they were monitored when the government did not provide them with commodities. Staff at the health facilities were more likely to have received malaria guidelines and case management training due to the ongoing MFT and MiMBa projects, which excluded drug outlets. During the roll-out of the MFT project, training was limited to facility-in-charges from health centres and dispensaries in addition to pharmacists from district and referral hospitals. It was expected that the trained personnel would sensitize other healthcare workers in their facilities. However, industrial action by providers in public health facilities during the period of the MFT roll-out may have hampered sensitization activities. In expanding MFT to other malaria-endemic areas in Kenya or other sub-Saharan African countries, consideration should be given to adequate healthcare providers’ sensitization while keeping in mind the possible disruptive effects of industrial action and other factors impeding the uptake of training.

Presumptive treatment of malaria in pregnant women with clinical symptoms emerged as a common practice. This has previously been documented in Kenya and other countries [32–36]. Women’s hesitancy to be tested with a malaria RDT due to association with HIV testing has not been described previously, though women’s concerns about fears of HIV testing hampering intermittent preventive treatment (IPTp) uptake in antenatal clinics have been documented [37]. There is a need to encourage providers to explain to patients the procedure for malaria diagnostic testing and distinguish it from HIV testing, in addition to a broader social and behavioural change strategy. Cases of needlestick injuries and a lack of gloves while performing diagnostic tests indicated a need for more training in infection prevention. Health facility providers had greater awareness than those from drug outlets that trimester was an important consideration when prescribing anti-malarials to pregnant women. Notwithstanding, drug outlet providers expressed challenges in providing pregnancy testing due to lack of pregnancy test kits, compromizing adherence to guidelines for treating malaria in early pregnancy. As over-the-counter medicines are the most popular choice for fever in Kenya [38], prioritizing pregnancy kits as essential commodities in drug outlets should be considered.

Healthcare provider preference has previously been found to outweigh knowledge of guidelines as a key driver in the type of anti-malarials prescribed [39]. In addition, the current study identified patient preference as a major consideration in prescribing anti-malarials, especially in the context of multiple first-line treatments. Patients were reported to prefer the once-daily dosing regimens of the newer artemisinin-based combinations to the twice-daily dosing of AL and thrice-daily seven-day quinine dosing. Providers preferred the blister packaging of artemisinin-based combinations for its ease in dispensing and perception that the labelling of daily doses contributed to improved adherence—though this was prescriber and not patient-reported. However, a preference for individual blister packaging of anti-malarial tablets by providers and pregnant women has previously been documented in several sub-Saharan African countries [40].

Drug stock-outs were noted to be a challenge. To alleviate this, some health facilities relied on their funds collected from patient fees to procure anti-malarials, while in some cases, drugs were redistributed between facilities. Quinine was reported to be most affected by stock-outs, even in health facilities, for various reasons: (1) providers reported it to be a slow-moving commodity that was rarely prescribed, (2) it was associated with many side effects such as the ringing of the ears [41]), bitter taste and premature uterine contractions (for intravenous doses), and (3) the regimen of three times daily for seven days for oral quinine was unfavourable to patients. As a result, providers prescribed AL instead of quinine, demonstrating poor adherence to the treatment policy at the time of the study, as also documented in a systematic review and meta-analysis [33]. It is worth noting that since the study was conducted, the safety of AL in the first trimester has been reviewed, and the WHO has since updated its treatment guidelines to recommend the use of AL over quinine, a policy also implemented in Kenya [6, 7, 42].

Cost was a universal factor driving the choice of drugs prescribed, despite government efforts to make anti-malarials free of charge in health facilities. Due to persistent stock-outs, the lack of free anti-malarial drugs in health facilities drove patients to buy the medicines they could afford in drug outlets. Particularly concerning was that drug outlets sold incomplete treatment courses to patients unable to afford the full dose. The National Malaria Control Programme would do well to ensure a regular supply of anti-malarials through timely procurement and distribution, as well as regulation of drug outlets to ensure adequate stocks. Consideration should be given to providing commodities—anti-malarials, RDTs and pregnancy kits—to drug outlets at a subsidized price. Healthcare provider access to anti-malarials and correct prescription practices are critical to correct case management and particularly relevant when introducing new therapies [43–45]. The in-depth interviews with providers in Western Kenya’s health facilities and drug outlets provide useful information to improve malaria case management and understand the challenges in implementing multiple first-line therapies.

This study has a few strengths. First, the study sample included public, private and faith-based facilities and drug outlet providers. Second, the inclusion of health managers raised important governance and regulatory issues. A study limitation is that patients were not included; thus, provider perceptions of pregnant women’s preferences and choices were not confirmed. One of the interviewers was a field officer with the MiMBa study and was known to some of the healthcare providers interviewed. However, no differences were noted between responses from healthcare providers known and unknown to him.

## Conclusion

The findings revealed a lack of knowledge of national guidelines and training for treatment of malaria in pregnancy among healthcare providers in drug outlets and private health facilities. To achieve its 2019–2023 strategic framework goals, it is important for the National Malaria Control Programme to prioritize training, supervision and regulation of drug outlet providers while consolidating the gains made in health facilities and increasing awareness of the safety of anti-malarials in pregnancy. In addition, a steady supply of malaria commodities and pregnancy detection kits is critical to ensuring proper case management.

## Data Availability

The transcripts analysed during the study are available from the authors on reasonable request. Interested researchers should contact the corresponding author on the email provided.

## Abbreviations

ANC: Antenatal care
ACT: Artemisinin-based combination therapy
AL: Artemether-lumefantrine
CHVs: Community health volunteers
COREQ: Consolidated criteria for reporting qualitative research
HIV: Human immunodeficiency virus
IPTp: Intermittent preventive treatment of malaria in pregnancy
MFT: Multiple first-line therapies
RDTs: Malaria rapid diagnostic tests
WHO: World Health Organization
WOCBA: Women-of-childbearing-age
